# Short-term outcome for high-risk patients after esophagectomy

**DOI:** 10.1093/dote/doac028

**Published:** 2022-06-21

**Authors:** Victor D Plat, Wessel T Stam, Boukje T Bootsma, Jennifer Straatman, Thomas Klausch, David J Heineman, Donald L van der Peet, Freek Daams, Dutch Upper GI Cancer Audit Group

**Affiliations:** Department of Gastrointestinal Surgery, Amsterdam UMC, VU University Medical Center, Amsterdam, The Netherlands; Department of Gastrointestinal Surgery, Amsterdam UMC, VU University Medical Center, Amsterdam, The Netherlands; Department of Gastrointestinal Surgery, Amsterdam UMC, VU University Medical Center, Amsterdam, The Netherlands; Department of Gastrointestinal Surgery, Amsterdam UMC, VU University Medical Center, Amsterdam, The Netherlands; Department of Epidemiology and Biostatistics, Amsterdam UMC, VU University Medical Center, Amsterdam, The Netherlands; Department of Gastrointestinal Surgery, Amsterdam UMC, VU University Medical Center, Amsterdam, The Netherlands; Department of Cardiothoracic Surgery, Amsterdam UMC, VU University Medical Center, Amsterdam, The Netherlands; Department of Gastrointestinal Surgery, Amsterdam UMC, VU University Medical Center, Amsterdam, The Netherlands; Department of Gastrointestinal Surgery, Amsterdam UMC, VU University Medical Center, Amsterdam, The Netherlands

**Keywords:** Charlson comorbidity index, mortality, population-based, propensity score matching, transhiatal esophagectomy, transthoracic esophagectomy

## Abstract

Transthoracic esophagectomy (TTE) for esophageal cancer facilitates mediastinal dissection; however, it has a significant impact on cardiopulmonary status. High-risk patients may therefore be better candidates for transhiatal esophagectomy (THE) in order to prevent serious complications. This study addressed short-term outcome following TTE and THE in patients that are considered to have a higher risk of surgery-related morbidity. This population-based study included patients who underwent a curative esophagectomy between 2011 and 2018, registered in the Dutch Upper GI Cancer Audit. The Charlson comorbidity index was used to assign patients to a low-risk (score ≤ 1) and high-risk group (score ≥ 2). Propensity score matching was applied to produce comparable groups between high-risk patients receiving TTE and THE. Primary endpoint was mortality (in-hospital/30-day mortality), secondary endpoints included morbidity and oncological outcomes. Additionally, a matched subgroup analysis was performed, including only cervical reconstructions. Of 5,438 patients, 945 and 431 high-risk patients underwent TTE and THE, respectively. After propensity score matching, mortality (6.3 vs 3.3%, *P* = 0.050), overall morbidity, Clavien-Dindo ≥ 3 complications, pulmonary complications, cardiac complications and re-interventions were significantly more observed after TTE compared to THE. A significantly higher mortality after TTE with a cervical reconstruction was found compared to THE (7.0 vs. 2.2%, *P* = 0.020). Patients with a high Charlson comorbidity index predispose for a complicated postoperative course after esophagectomy, this was more outspoken after TTE compared to THE. In daily practice, these outcomes should be balanced with the lower lymph node yield, but comparable positive node count and radicality after THE.

## INTRODUCTION

Trimodality therapy represents the gold standard for potentially curable esophageal cancer.^1^ However, the most appropriate surgical approach is a subject of ongoing debate. In the open and monomodal treatment era, randomized evidence showed that an open transthoracic esophagectomy (TTE) was associated with significantly more pulmonary complications compared to a transhiatal esophagectomy (THE).^2^ A recent population-based study solidified these findings and observed an additional increase in short-term mortality rates after TTE.^3^ The potential benefit of a TTE entails a more extensive thoracic lymphadenectomy, which may improve overall oncological survival.^3–5^ This survival advantage was confirmed in patients with locally advanced tumors and nodal tumor spread.^6^

Next to tumor specifics (histology, stage, location and nodal spread), other factors such as previous thoracic surgery (e.g. pleurectomy or pulmonary surgery), neoadjuvant chemotherapy, neoadjuvant radiation field and preference of the surgeon, determine the surgical approach. However, the patient’s physical status remains the single most important factor in the decision process. Frail patients with a poor pulmonary or cardiac status are considered at high risk for adverse outcomes after esophagectomy.^7–9^ These patients can be identified using the Charlson comorbidity index, which is a validated risk assessment tool.^10^^,^^11^ High-risk patients could be better candidates for THE in order to prevent serious postoperative complications,^12–14^ but at the same time denying them the established oncological benefits of TTE.^4^^,^^5^

Recently, postoperative outcomes have improved with the introduction of minimally invasive surgery and neoadjuvant chemoradiotherapy.^1^^,^^15^ The debate on which procedure is most suitable for high-risk patients is therefore reopened as data on short-term postoperative outcomes in the minimally invasive and trimodality era are currently lacking. To address the question which approach is preferred in patients at high-risk for postoperative complications, this study first compared short-term mortality and postoperative outcomes between low- and high-risk patients following TTE or THE separately. Subsequently, a propensity score matched analysis was performed to assess the difference in short-term mortality and postoperative complication profiles between TTE and THE, followed by a subgroup analysis including only cervical reconstructions.

## METHODS

For this national comparative cohort study, data were extracted from the Dutch Upper GI Cancer Audit (DUCA) registers. In the Netherlands, it is mandatory for all hospitals to register the results of surgical procedures for esophageal and gastric cancer with the intention to cure in this database. The DUCA data have been verified regarding its completeness and accuracy of postoperative complication registration, showing that the data are representative for the Dutch esophageal cancer patient population.^16^ This study was reported according to the STROBE guidelines^17^ and its protocol approved by the scientific committee of the DUCA. No informed consent or ethical approval was required.

### Eligibility criteria

Patients undergoing an open or minimally invasive TTE or THE for esophageal cancer in the Netherlands between January 2011 and December 2018 were identified from the DUCA registry. Patients who underwent an emergency procedure, procedure without the intention to cure, in whom no gastric conduit (e.g. colon interposition, roux-en-Y jejunostomy or jejunal interposition) was created or continuity was not restored via primary anastomosis (cervical anastomosis after THE (Orringer procedure)^18^ and cervical (McKeown procedure)^19^ or intrathoracic anastomosis (Ivor Lewis procedure)^20^ after TTE) were not eligible for inclusion. Patients with missing data on eligibility criteria or relevant patient characteristics were excluded. Race or ethnicity data were not available.

### Group selection

The Charlson comorbidity index^10^ modified by Deyo’s coding algorithm^21^ was used to divide the patients into a low-risk and high-risk groups. The Charlson comorbidity index categorizes patients into six classes (0, 1, 2, 3, 4 or ≥5) based on the existence of comorbidities. Every comorbid condition scores one, two or six points depending on their predetermined effect. Points of all comorbid conditions were added to calculate the index score. Patients with a Charlson comorbidity index score of ≥2 were considered high risk, subsequently patients with a score of ≤1 were considered low-risk. The cut-off value of two was chosen based on the findings of previous studies investigating the use of the Charlson comorbidity index in esophageal cancer patients.^11^^,^^22–24^ Most comorbidities were separately registered in the DUCA database. However, dementia was not an individual parameter and was combined with parkinsonism in the registry. Chronic obstructive pulmonary disease, asthma and lung fibrosis were recorded individually and were taken together as chronic pulmonary disease. Rheumatologic disease, sarcoidosis, Besnier Boeck, systemic lupus erythematosus, scleroderma and vasculitis were individual parameters and were taken together as rheumatologic disease. Moderate to severe kidney disease was defined as chronic kidney disease with a creatinine level above 110 μmol/L or dialysis dependent renal failure. Liver disease was registered as a single parameter and not graded according to severity; therefore, it was decided to include liver disease as a single category assigned with two points. This resulted in 16 categories, the comorbid conditions and their assigned scores are summarized in Table 1.

### Outcome measures and definitions

The primary outcome was postoperative mortality defined as mortality during initial hospital admission or within 30 days after surgery.

Overall postoperative morbidity was analyzed as a secondary endpoint and was divided in surgical complications (anastomotic leakage and intra-abdominal or thoracic abscesses), systemic complications (pulmonary or cardiac) and re-interventions (surgical, endoscopic or radiological re-interventions). Complications were defined according to standards of the DUCA and graded according to Clavien-Dindo.^25^ Complications with a Clavien-Dindo score of ≥3 were considered severe complications. Anastomotic leakage was defined as a clinical, endoscopic or radiological diagnosed defect of the gastric conduit, anastomosis or staple line. Intra-abdominal and thoracic abscesses were defined as pus-containing non pre-existent cavities requiring additional drainage. Pulmonary complications consisted of pneumonia (defined according to the American Thoracic Society and Infectious Diseases Society of America as a new lung infiltrate on radiological imaging and two out of the following three criteria: fever, leukocytosis or purulent sputum),^26^ pleural effusion requiring additional drainage, pneumothorax requiring intervention, atelectasis, acute respiratory distress syndrome, persisting air leakage (present >10 days after surgery) and the need for re-intubation. Cardiac complications were defined as complications that necessitated treatment or cardiopulmonary resuscitation including myocardial infarction, supraventricular arrhythmia, congestive heart failure and cardiac arrest. A severely complicated clinical course was defined as a postoperative morbidity leading to a prolonged hospital stay (>21 days), re-intervention or mortality. Failure to rescue was defined as postoperative mortality among patients with a complicated clinical course. General recovery was evaluated by initial hospital stay, intensive/medium care unit (ICU) stay and number of readmissions within 30 days after surgery. Finally, oncological quality indicators of the surgical resection specimen were analyzed, including R0 resection rate (defined as a microscopically margin-negative resection) and (positive) lymph node yield.

**Table 1:** Charlson comorbidity index scoring system

Comorbidity condition	Assigned score
Myocardial infarction	1
Congestive cardiac insufficiency	
Peripheral vascular disease	
Cerebrovascular disease	
Dementia or parkinsonism	
Chronic pulmonary disease	
Rheumatologic disease	
Well-regulated diabetes	
Peptic ulcer disease	
	
Liver disease	2
Hemiplegia	
Moderate to severe kidney disease	
Diabetes with end-organ damage	
Any curatively treated malignancy	
	
HIV or AIDS	6
Any palliative treated malignancy	

AIDS indicates acquired immunodeficiency syndrome; HIV, human immunodeficiency virus

### Statistical analysis

Statistical analysis was performed using SPSS (version 26. IBM Corp, Armonk, NY). A two-sided *P*-value of <0.05 was considered to be statistically significant. For continuous data, median and interquartile ranges (IQR) are given; other characteristics are reported using frequencies and percentages. The Charlson comorbidity index score was calculated and patients were designated as low-risk or high-risk based on a score cut-off value of two. Comparisons between high-risk versus low-risk patients, stratified for type of procedure, were performed using Pearson chi-square test or Fisher’s exact test (in case of small cell counts) for categorical variables. The Mann–Whitney *U* test was used for continuous variables. The average causal effect between high-risk versus low-risk patients was estimated for TTE and THE separately. First, propensity scores, stratified for type of surgery, were computed for each patient using logistic regression, incorporating main effects of all relevant covariates in the equation. Covariates were considered relevant when potentially effecting the primary outcome and not derived from the Charlson comorbidity index: age, body mass index (BMI), gender, tumor histology, tumor location (distance from the incisors), cT-stage, cN-stage, neoadjuvant therapy and previous abdominal or thoracic surgery. Second, patients were classified into five strata using the quintiles of propensity scores. Finally, average effects were estimated using a multivariate forward stepwise logistic regression analysis, including propensity strata and previously mentioned covariates as predictors.^27^ An additional analysis was performed to address the difference between minimally invasive and open procedures in high-risk patients, stratified for operative technique.

### Propensity score matching

Propensity score matching was used to balance differences in preoperative factors between high-risk patients who underwent TTE or THE and between a subgroup of patients who underwent McKeown or THE. This procedure was designed for minimizing the effects of confounding, creating two comparable groups. Before estimation of the propensity scores, patients with tumors located in the upper and middle third of the esophagus were excluded as THE is only performed in patients with tumors of the lower esophagus or gastroesophageal junction. A propensity score was computed for each patient using logistic regression, incorporating main effects of all relevant covariates in the equation. Covariates were considered relevant when available before surgery and/or used to determine the surgical approach: age, body mass index (BMI), gender, Charlson comorbidity index score, American Society of Anesthesiologists (ASA) score, tumor histology, tumor location (distance from the incisors), cT-stage, cN-stage, neoadjuvant therapy and previous abdominal or thoracic surgery. Covariate categories were combined in case of insufficient cell counts. After estimation of the propensity scores, TTE patients were matched to THE patients using 1:1 nearest-neighbor matching without replacement. Presented estimates represent the causal risk difference and odds ratio (OR) if THE treated patients had been treated by TTE instead. A subgroup analysis was performed, including only cervical reconstructions, using propensity scores to match THE patients to McKeown patients using the same methods. It is recommended to use a maximum allowable difference between two matched participants defined by the logit of the propensity score using calipers of width equal to 0.2 of the standard deviation of the logit of the propensity score.^28^ Standardized mean differences (SMD) were estimated for all covariates before and after matching, according to recent recommendations a SMD of 0.10 or more was considered indicative of imbalance.^29^ Univariate logistic regression analysis or Mann–Whitney *U* tests were performed in the unmatched and matched groups to estimate causal effects. Two additional subgroup analyses were performed: patients with adenocarcinoma’s after TTE versus THE and patients after a minimally invasive TTE versus open THE. Propensity score matching was conducted using R 3.5 open-source software (MatchIt’ and ‘optmatch’) to SPSS (version 26. IBM Corp, Armonk, NY).

## RESULTS

### Patient selection

**Fig. 1:**
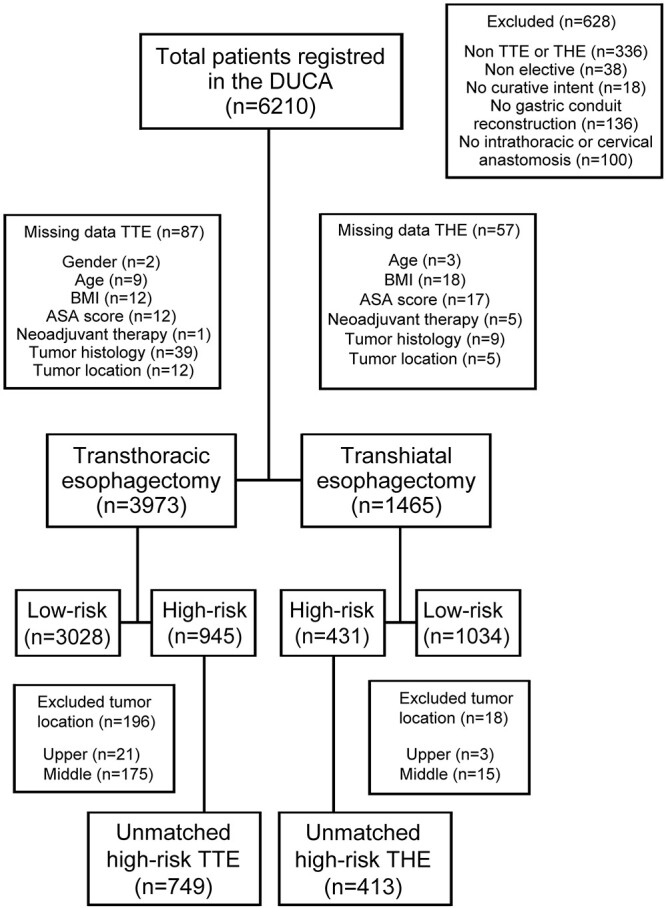
Flowchart of patient distribution. TTE, transthoracic esophagectomy; THE, transhiatal esophagectomy.

Between January 2011 and December 2018, 6,210 patients with esophageal cancer were registered in the DUCA. An eligible TTE was performed in 4,060 patients, no data were missing in 3,973 patients. An eligible THE with curative intent was performed in 1,522 patients, of which 1,465 patients had no missing data. Patients were divided into a low-risk and high-risk groups based on the Charlson comorbidity index score. A flow chart of the patient selection and distribution is shown in Figure 1.

### Group characteristics

Significant differences between high-risk and low-risk patients in both groups were observed (Table 2), including age, ASA score, clinical T stage, neoadjuvant therapy and history of abdominal or thoracic surgery. In the TTE group, the clinical N stage and BMI differed significantly between the two risk categories. Within the THE group, a minimally invasive approach was more often performed in high-risk patients.

**Table 2:** Baseline characteristics of low-risk (CCI ≤ 1) and high-risk patients (CCI ≥ 2) stratified for type of surgery

Characteristics	TTE *n* = 3,973			THE *n* = 1,465		
	High-risk *n* = 945	Low-risk *n* = 3,028	*P*-value^^*^^	High-risk *n* = 431	Low-risk *n* = 1,034	*P*-value^^*^^
Age, years	68 (63–72)	65 (58–70)	*<0.001* ^†^	70 (64–75)	66 (59–72)	*<0.001* ^†^
BMI, kg/m^2^	25.7 (23.2–28.7)	25.4 (23.0–28.1)	0.159^†^	26.1 (23.5–29.3)	25.7 (23.2–28.7)	0.270^†^
Gender			*0.011*			0.415
Male	697 (73.8)	2,354 (77.7)		337 (78.2)	828 (80.1)	
CCI score			—			—
0	—	2,070 (68.4)		—	664 (64.2)	
1	—	958 (31.6)		—	370 (35.8)	
2	586 (62.0)	—		261 (60.6)	—	
3	249 (26.3)	—		103 (23.9)	—	
4	60 (6.3)	—		44 (10.2)	—	
≥5	50 (5.3)	—		23 (5.3)	—	
ASA-classification			*<0.001*			*<0.001*
I	60 (6.3)	588 (19.4)		30 (7.0)	195 (18.9)	
II	554 (58.6)	1,923 (63.5)		217 (50.3)	634 (61.3)	
III	327 (34.6)	510 (16.8)		173 (40.1)	204 (19.7)	
IV	4 (0.4)	7 (0.2)		11 (2.6)	1 (0.1)	
Tumor histology			*<0.001*			*0.039*
AC	651 (68.9)	2,316 (76.5)		357 (82.8)	907 (87.7)	
SCC	266 (28.1)	643 (21.2)		65 (15.1)	108 (10.4)	
Other	28 (3.0)	69 (2.3)		9 (2.1)	19 (1.8)	
Tumor location			*0.042*			0.347
Proximal	21 (2.2)	43 (1.4)		3 (0.7)	5 (0.5)	
Middle	175 (18.5)	472 (15.6)		15 (3.5)	22 (2.1)	
Distal	603 (63.8)	1,999 (66.0)		267 (61.9)	626 (60.5)	
GEJ	146 (15.4)	514 (17.0)		146 (33.9)	381 (36.8)	
From the incisors, cm	34 (30–37)	35 (31–38)	*<0.001* ^†^	36 (34–38)	36 (34–38)	0.192^†^
cT-stage			*0.023*			*0.001*
cT0	5 (0.5)	15 (0.5)		6 (1.4)	1 (0.1)	
cT1	65 (6.9)	126 (4.2)		19 (4.4)	51 (4.9)	
cT2	175 (18.5)	540 (17.8)		95 (22.0)	193 (18.7)	
cT3	641 (67.8)	2,130 (70.3)		279 (64.7)	723 (69.9)	
cT4	26 (2.8)	103 (3.4)		7 (1.6)	31 (3.0)	
cTx	33 (3.5)	114 (3.8)		25 (5.8)	35 (3.4)	
cN-stage			*0.005*			*0.001*
cN0	369 (39.0)	1,025 (33.9)		192 (44.5)	356 (34.4)	
cN1	356 (37.7)	1,227 (40.5)		153 (35.5)	423 (40.9)	
cN2	157 (16.6)	583 (19.3)		52 (12.1)	189 (18.3)	
cN3	21 (2.2)	96 (3.2)		11 (2.6)	21 (2.0)	
cNx	42 (4.4)	97 (3.2)		23 (5.3)	45 (4.4)	
Neoadjuvant therapy			*<0.001*			*<0.001*
Chemotherapy	42 (4.4)	169 (5.6)		33 (7.7)	97 (9.4)	
Radiotherapy	2 (0.2)	5 (0.2)		0 (0.0)	0 (0.0)	
CRT	799 (84.6)	2,667 (88.1)		322 (74.7)	851 (82.3)	
None	102 (10.8)	187 (6.2)		76 (17.6)	86 (8.3)	
Previous abdominal or thoracic surgery						
Yes	400 (42.3)	748 (24.7)	*<0.001*	178 (41.3)	275 (26.6)	*<0.001*
Approach			*0.819*			*0.001*
Open	160 (16.9)	532 (17.6)		257 (59.6)	714 (69.1)	
MI abdomen	43 (4.6)	118 (3.9)		174 (40.4)	320 (30.9)	
MI thorax	26 (2.8)	84 (2.8)		—	—	
MIE	716 (75.8)	2,294 (75.8)		—	—	
Anastomosis			0.424			—
Intrathoracic	514 (54.4)	1,602 (52.9)		0 (0)	0 (0)	
Cervical	431 (45.6)	1,426 (47.1)		431 (100)	1,034 (100)	

Data are *n* (%) or median (IQR). *P*-values < 0.05 are denoted in italic. AC indicates adenocarcinoma; ASA, American Association of Anesthesiologists; BMI, body mass index; CCI, Charlson Comorbidity Index; CRT, chemoradiotherapy; GEJ, gastroesophageal junction; IQR, interquartile range; MI, minimally invasive; MIE, minimally invasive esophagectomy; SCC, squamous cell carcinoma; THE, transhiatal esophagectomy; TTE, transthoracic esophagectomy.

^*^ *P*-value calculated by χ2 test

^†^Mann–Whitney *U* test.

### Postoperative outcomes in high-risk versus low-risk patients stratified for operative technique

Postoperative mortality rates were significantly higher in high-risk TTE patients (5.9 vs. 2.8%, OR: 2.040, *P* < 0.001) compared to low-risk TTE patients (Table 3). Postoperative overall morbidity (70.7 vs. 61.9%, OR: 1.378, *P* < 0.001), Clavien-Dindo score ≥ 3 complications (33.0 vs. 27.6%, OR: 1.252, *P* = 0.007), failure to rescue rates (15.3 vs. 8.7%, OR: 1.754, *P* = 0.004), pulmonary complications (41.6 vs. 32.1%, OR: 1.417, *P* < 0.001), re-intervention rates (30.7 vs. 25.8%, OR: 1.238, *P* = 0.012) and readmission rates (16.9 vs. 14.2%, OR: 1.231, *P* = 0.047) were significantly higher in high-risk TTE patients. Furthermore, there was a significant delay in hospital (*P* < 0.001) and ICU discharge (*P* < 0.001). Besides significantly less positive lymph nodes in the resected specimen of high-risk TTE patients (*P* = 0.004), no oncological outcome differences (lymph node yield and R0 resection) were observed.

**Table 3:** Postoperative outcome measures in high-risk patients versus low-risk patients stratified for type of surgery

Postoperative outcome	TTE *n* = 3,973				THE *n* = 1,465			
	High-risk *n* = 945	Low-risk *n* = 3,028	Adjusted OR (95% CI)^*^	*P*-value^*^	High-risk *n* = 431	Low-risk *n* = 1,034	Adjusted OR (95% CI)^*^	*P*-value^*^
Mortality	56 (5.9)	85 (2.8)	2.040 (1.423–2.928)	*<0.001*	15 (3.5)	25 (2.4)	1.298 (0.660–2.553)	0.449
Morbidity	668 (70.7)	1,875 (61.9)	1.378 (1.169–1.624)	*<0.001*	272 (63.1)	561 (54.3)	1.212 (0.948–1.549)	0.125
Complication CD ≥3	312 (33.0)	837 (27.6)	1.252 (1.064–1.473)	*0.007*	110 (25.5)	163 (15.8)	1.578 (1.180–2.112)	*0.002*
Severely complicated course	367 (38.8)	942 (31.1)	1.318 (1.126–1.542)	*0.001*	129 (29.9)	213 (20.6)	1.376 (1.047–1.809)	*0.022*
Failure to rescue^‡^	56 (15.3)	82 (8.7)	1.754 (1.195–2.574)	*0.004*	15 (11.6)	22 (10.3)	1.271 (0.581–2.781)	0.549
Surgical complications								
Anastomotic leakage	197 (20.8)	580 (19.2)	1.082 (0.897–1.305)	0.410	108 (25.1)	177 (17.1)	1.528 (1.146–2.038)	*0.004*
Abscess	14 (1.5)	36 (1.2)	1.160 (0.611–2.203)	0.650	4 (0.9)	10 (1.0)	0.959 (0.299–3.075)	0.944
Systemic complications								
Pulmonary	393 (41.6)	972 (32.1)	1.417 (1.212–1.656)	*<0.001*	128 (29.7)	235 (22.7)	1.245 (0.954–1.626)	0.107
Cardiac	187 (19.8)	462 (15.3)	1.204 (0.989–1.464)	0.064	60 (13.9)	103 (10.0)	1.224 (0.857–1.748)	0.267
Re-interventions	290 (30.7)	780 (25.8)	1.238 (1.049–1.462)	*0.012*	94 (21.8)	144 (13.9)	1.565 (1.152–2.125)	*0.004*
Recovery								
Hospital stay, days	14 (10–25)	12 (9–20)	—	*<0.001* ^†^	12 (9–19)	10 (8–15)	—	*<0.001* ^*†*^
ICU stay, days	2 (1–6)	2 (1–4)	—	*<0.001* ^†^	2 (1–4)	1 (1–3)	—	*0.008* ^*†*^
Readmission	160 (16.9)	430 (14.2)	1.231 (1.002–1.511)	*0.047*	61 (14.2)	103 (10.0)	1.450 (1.017–2.067)	*0.040*
Pathology								
R0	906 (95.9)	2,882 (95.1)	1.030 (0.705–1.505)	0.879	413 (95.8)	969 (93.7)	1.471 (0.832–2.600)	0.185
Lymph nodes	21 (16–28)	22 (17–29)	—	0.053^†^	14 (10–19)	15 (10–19)	—	0.278^†^
Positive lymph nodes	0 (0–1)	0 (0–1)	—	*0.004* ^†^	0 (0–1)	0 (0–2)	—	*0.015* ^†^

Data are *n* (%), median (IQR) or adjusted OR (95% CI). *P*-values < 0.05 are denoted in italic. ASA indicates American Association of Anesthesiologists; CI, confidence interval; ICU, intensive care unit; IQR, interquartile range; OR, odds ratio; THE, transhiatal esophagectomy; TTE, transthoracic esophagectomy.

^*^Low-risk used as reference, *P*-value calculated by multivariate logistic regression analysis.

^†^Mann–Whitney *U* test.

^‡^Mortality within patients with a severely complicated clinical course.

In THE patients, no significant difference in mortality between high and low-risk patients was observed (*P* = 0.449). High-risk patients had a higher rate of Clavien-Dindo score ≥3 complications (25.5 vs. 15.8%, OR: 1.578, *P* = 0.002), anastomotic leakage (25.1 vs. 17.1%, OR: 1.528, *P* = 0.004), re-interventions (21.8 vs. 13.9%, OR: 1.565, *P* = 0.004) and readmission (14.2 vs. 10.0%, OR: 1.450, *P* = 0.040) compared to low-risk patients. In addition, hospital discharge (*P* < 0.001) and ICU discharge (*P* = 0.008) were significantly delayed. Significantly less positive lymph nodes were found in the resected specimen of high-risk THE patients (*P* = 0.015), no differences in general lymph node yield and R0 resection were observed.

### Propensity score matched high-risk patients

Propensity score matching was used to match 399 high-risk patients who underwent TTE to 399 high-risk patients who underwent THE. The absolute standardized differences were less than 0.10 for all preoperative variables included in the propensity score, indicating a successful match (Table 4). No suitable match was found in 14 THE patients. Compared to those who were matched, the unmatched THE patients were significantly older (median age: unmatched 78 years, matched 70 years) and had higher Charlson comorbidity index scores (Charlson comorbidity index of 4: unmatched 38%, matched 9%). Distance to incisors (unmatched 40, matched 36). Unmatched patients had a lower tumor level (median distance from incisors: 40 centimeters vs. 36 centimeters) and underwent less neoadjuvant chemoradiotherapy (unmatched 7%, matched 78%).

**Table 4:** Baseline characteristics of high-risk after TTE and THE before and after propensity score matching

Characteristics	Unmatched cohort high-risk *n* = 1,162			Matched cohort high-risk *n* = 798		
	TTE *n* = 749	THE *n* = 413	SMD	TTE *n* = 399	THE *n* = 399	SMD
Age, years	68 (63–72)	70 (64–75)	0.23	70 (65–73)	70 (64–74)	0.00
BMI, kg/m^2^	26.2 (23.6–28.9)	26.1 (23.5–29.2)	0.02	26.3 (23.7–28.9)	26.1 (23.5–29.1)	0.02
Gender						
Male	607 (81.0)	327 (79.2)	0.05	315 (78.9)	317 (79.4)	0.01
CCI score						
2	257 (61.0)	251 (60.8)	0.00	246 (61.7)	244 (61.2)	0.01
3	204 (27.2)	98 (23.7)	0.08	96 (24.1)	96 (24.1)	0.00
4	47 (6.3)	42 (10.2)	0.14	38 (9.5)	37 (9.3)	0.01
≥5	41 (5.5)	22 (5.3)	0.01	19 (4.8)	22 (5.5)	0.03
ASA-classification						
I	44 (5.9)	28 (6.8)	0.04	28 (7.0)	27 (6.8)	0.04
II	431 (57.5)	212 (51.3)	0.12	214 (53.6)	207 (51.9)	0.03
III–IV	274 (36.6)	173 (41.9)	0.11	157 (39.3)	165 (41.4)	0.04
Tumor histology						
AC	611 (81.6)	350 (84.7)	0.08	335 (84.0)	336 (84.2)	0.01
SCC	115 (15.4)	54 (13.1)	0.07	57 (14.3)	54 (13.5)	0.02
Other	23 (3.1)	9 (2.2)	0.06	7 (1.8)	9 (2.3)	0.04
Tumor location						
Distal^*^	603 (80.5)	267 (64.6)	—	312 (78.2)	263 (65.9)	—
GEJ^*^	146 (19.5)	146 (35.4)	—	87 (21.8)	136 (34.1)	—
From the incisors, cm	35 (33–38)	36 (34–38)	0.25	35 (34–38)	36 (34–38)	0.04
cT-stage						
cT0–1	56 (7.5)	23 (5.6)	0.08	29 (7.3)	23 (5.8)	0.06
cT2	137 (18.3)	92 (22.3)	0.10	80 (20.1)	88 (22.1)	0.05
cT3–4	529 (70.6)	277 (67.1)	0.08	274 (68.7)	268 (67.2)	0.03
cTx	27 (3.6)	21 (5.1)	0.07	16 (4.0)	20 (5.0)	0.05
cN-stage						
cN0	297 (39.7)	185 (44.8)	0.10	178 (44.6)	177 (44.4)	0.01
cN1	282 (37.7)	149 (36.1)	0.03	144 (36.1)	145 (36.3)	0.00
cN2–3	136 (18.2)	60 (14.5)	0.10	60 (15.0)	58 (14.5)	0.01
cNx	34 (4.5)	19 (4.6)	0.00	17 (4.3)	19 (4.8)	0.02
Neoadjuvant therapy						
None	77 (10.3)	69 (16.7)	0.19	60 (15.0)	58 (14.5)	0.01
Chemotherapy	37 (4.9)	33 (8.0)	0.13	31(7.8)	31 (7.8)	0.00
CRT or radiotherapy	635 (84.8)	311 (75.3)	0.24	308 (77.2)	310 (77.7)	0.01
Previous abdominal or thoracic surgery						
Yes	312 (41.7)	170 (41.2)	0.01	167 (41.9)	164 (41.1)	0.02
Approach^*^						
Open	116 (15.5)	246 (59.6)	—	64 (16.0)	235 (58.9)	—
MI abdomen	35 (4.7)	167 (40.4)	—	21 (5.3)	164 (41.1)	—
MI thorax	13 (1.7)	—	—	6 (1.5)	—	—
MIE	585 (78.1)	—	—	308 (77.2)	—	—
Anastomosis^*^						
Intrathoracic	467 (62.3)	0 (0)	—	253 (63.4)	0 (0)	—
Cervical	282 (37.7)	413 (100)	—	146 (36.6)	399 (100)	—

Data are *n* (%) or median (IQR). AC indicates adenocarcinoma; ASA, American Association of Anesthesiologists; BMI, body mass index; CCI, Charlson Comorbidity Index; CRT, chemoradiotherapy; GEJ, gastroesophageal junction; IQR, interquartile range; MI, minimally invasive; MIE, minimally invasive esophagectomy; SMD, standardized mean difference; SCC, squamous cell carcinoma; THE, transhiatal esophagectomy; TTE, transthoracic esophagectomy.

^*^Variables were not used in propensity score matching.

### Outcomes after matching

A significantly higher postoperative mortality was observed in high-risk patients after TTE compared to THE (6.3 vs. 3.3%, OR: 1.984, *P* = 0.050). Furthermore, significantly more overall morbidity (72.2 vs. 63.4%, OR: 1.497, *P* = 0.008), Clavien-Dindo score ≥3 complications (33.8 vs. 24.6%, OR 1.571, *P* = 0.004), pulmonary complications (41.6 vs. 28.8%, OR: 1.759, *P* < 0.001), cardiac complications (20.1 vs. 14.5%, OR: 1.474, *P* = 0.040) and re-interventions (30.8 vs. 21.3% OR: 1.646, *P* = 0.002) were observed. Hospital discharge (*P* = 0.001) and ICU discharge (*P* = 0.002) were delayed after TTE compared to THE. Failure to rescue rates (15.8 vs. 11.2%, *P* = 0.277) and anastomotic leakage rates (21.8 vs. 24.6%, *P* = 0.356) were not significantly different between TTE and THE. TTE yielded significantly more lymph nodes (*P* < 0.001), yet there was no difference in the median number of positive lymph nodes or R0 resection rate. All primary and secondary outcome measures of high-risk patients after TTE and THE before and after propensity score matching are displayed in Table 5.

**Table 5:** Postoperative outcome measures of high-risk patients following TTE and THE before and after propensity score matching

Postoperative outcome	Unmatched cohort high-risk *n* = 1,162				Matched cohort high-risk *n* = 798			
	TTE *n* = 749	THE *n* = 413	OR (95% CI)^^*^^	*P*-value^^*^^	TTE *n* = 399	THE *n* = 399	OR (95% CI)^^*^^	*P*-value^^*^^
Mortality	44 (5.9)	14 (3.4)	1.779 (0.963–3.286)	0.066	25 (6.3)	13 (3.3)	1.984 (1.000–3.938)	*0.050*
Morbidity	524 (70.0)	261 (63.2)	1.356 (1.052–1.748)	*0.019*	288 (72.2)	253 (63.4)	1.497 (1.110–2.190)	*0.008*
Complication CD ≥3	231 (30.8)	102 (24.7)	1.360 (1.036–1.785)	*0.027*	135 (33.8)	98 (24.6)	1.571 (1.154–2.137)	*0.004*
Severely complicated course	272 (36.3)	121 (29.3)	1.376 (1.062–1.783)	*0.016*	158 (39.6)	116 (29.1)	1.599 (1.191–2.148)	*0.002*
Failure to rescue‡	44 (16.2)	14 (11.6)	1.475 (0.775–2.808)	0.237	25 (15.8)	13 (11.2)	1.489 (0.726–3.053)	0.277
Surgical complications								
Anastomotic leakage	150 (20.0)	102 (24.7)	0.764 (0.573–1.017)	0.065	87 (21.8)	98 (24.6)	0.856 (0.616–1.190)	0.356
Abscess	11 (1.5)	4 (1.0)	1.524 (0.482–4.817)	0.473	4 (1.0)	4 (1.0)	1.000 (0.248–4.027)	1.000
Systemic complications								
Pulmonary	304 (40.6)	122 (29.5)	1.629 (1.260–2.107)	*<0.001*	166 (41.6)	115 (28.8)	1.759 (1.311–2.361)	*<0.001*
Cardiac	153 (20.4)	60 (14.5)	1.510 (1.090–2.093)	*0.013*	80 (20.1)	58 (14.5)	1.474 (1.018–2.136)	*0.040*
Re-interventions	210 (28.0)	88 (21.3)	1.439 (1.083–1.912)	*0.012*	123 (30.8)	85 (21.3)	1.646 (1.195–2.268)	*0.002*
Recovery								
Hospital stay, days	13 (9–22)	12 (9–18)	—	*0.009* ^†^	14 (10–23)	12 (9–18)	—	*0.001* ^*†*^
ICU stay, days	2 (1–5)	2 (1–4)	—	*0.001* ^†^	2 (1–5)	2 (1–4)	—	*0.002* ^*†*^
Readmission	127 (17.0)	59 (14.3)	1.225 (0.876–1.713)	0.235	66 (16.5)	59 (14.8)	1.142 (0.779–1.674)	0.496
Pathology								
R0	719 (96.0)	396 (95.9)	1.029 (0.560–1.889)	0.927	381 (95.5)	382 (95.7)	0.942 (0.478–1.855)	0.863
Lymph nodes	21 (16–27)	14 (10–19)	—	*<0.001* ^†^	20 (16–27)	14 (10–19)	—	*<0.001* ^*†*^
Positive lymph nodes	0 (0–1)	0 (0–1)	—	0.311^†^	0 (0–1)	0.0 (0–1)	—	0.124^†^

Data are *n* (%), median (IQR) or OR (95% CI). *P*-values < 0.05 are denoted in italic. ASA indicates American Association of Anesthesiologists; CI, confidence interval; ICU, intensive care unit; IQR, interquartile range; OR, odds ratio; THE, transhiatal esophagectomy; TTE, transthoracic esophagectomy.

^*^THE used as reference, *P*-value calculated by logistic regression analysis.

^†^Mann–Whitney *U* test.

^‡^Mortality within patients with a severely complicated clinical course.

In a subgroup of patients with adenocarcinoma’s (335 patients after TTE vs. 336 patients after THE), mortality remained significantly higher after TTE compared to THE (5.7 vs. 2.4%, OR: 2.465, *P* = 0.032). This also applied to morbidity (72.2 vs. 63.4%, OR: 1.503, *P* = 0.017), pulmonary complications (42.2 vs. 28.9%, OR: 1.813, *P* < 0.001), re-interventions (31.9 vs. 20.8%, OR: 1.050, *P* = 0.001), delay in hospital discharge (*P* = 0.004) and delay in ICU discharge (*P* = 0.004).

### Subgroup analysis including cervical reconstructions

A propensity score matched subgroup analysis was performed matching 230 patients who underwent THE to 230 patients who underwent TTE with a cervical reconstruction (McKeown procedure). The absolute standardized differences between preoperative variables are depicted in Table S1. Logistic regression analysis revealed a significantly higher postoperative mortality rate in high-risk patients after McKeown compared to THE (7.0 vs. 2.2%, OR: 3.364, *P* = 0.020). Furthermore, significantly more overall morbidity (75.2 vs. 60.4%, OR: 1.987, *P* = 0.001), severely complicated clinical courses (40.4 vs. 26.5%, OR: 1.881, *P* = 0.002) and pulmonary complications (43.9 vs. 27.8%, OR: 2.031, *P* < 0.001) were observed. Hospital discharge (*P* = 0.001) was delayed after McKeown compared to THE. A transthoracic approach yielded significantly more lymph nodes (*P* < 0.001), however no differences in positive lymph nodes or R0 resection rates were observed. All postoperative outcome measures of high-risk patients following McKeown and THE before and after matching are summarized in Table 6.

**Table 6:** Postoperative outcome measures of high-risk patients following McKeown and THE before and after propensity score matching

Postoperative outcome	Unmatched cohort high-risk *n* = 695				Matched cohort high-risk *n* = 460			
	McKeown *n* = 282	THE *n* = 413	OR (95% CI)^^*^^	*P*-value^^*^^	McKeown *n* = 230	THE *n* = 230	OR (95% CI)^^*^^	*P*-value^^*^^
Mortality	21 (7.4)	14 (3.4)	2.293 (1.146–4.590)	*0.019*	16 (7.0)	5 (2.2)	3.364 (1.211–9.344)	*0.020*
Morbidity	214 (75.9)	261 (63.2)	1.833 (1.307–2.571)	*<0.001*	173 (75.2)	139 (60.4)	1.987 (1.333–2.962)	*0.001*
Complication CD ≥3	85 (30.1)	102 (24.7)	1.316 (0.938–1.846)	0.112	72 (31.3)	55 (23.9)	1.450 (0.961–2.188)	0.077
Severely complicated course	109 (38.7)	121 (29.3)	1.520 (1.104–2.094)	*0.010*	93 (40.4)	61 (26.5)	1.881 (1.269–2.788)	*0.002*
Failure to rescue^‡^	21 (19.3)	14 (11.6)	1.824 (0.877–3.795)	0.108	16 (17.6)	5 (8.2)	2.327 (0.805–6.728)	0.119
Surgical complications								
Anastomotic leakage	62 (22.0)	102 (24.7)	0.859 (0.600–1.231)	0.409	52 (22.6)	50 (21.7)	1.052 (0.677–1.633)	0.822
Abscess	4 (1.4)	4 (1.0)	1.471 (0.365–5.932)	0.587	3 (1.3)	3 (1.3)	1.000 (0.200–5.007)	1.000
Systemic complications								
Pulmonary	122 (43.3)	122 (29.5)	1.819 (1.325–2.496)	*<0.001*	101 (43.9)	64 (27.8)	2.031 (1.377–2.995)	*<0.001*
Cardiac	52 (18.4)	60 (14.5)	1.330 (0.886–1.998)	0.169	40 (17.4)	41 (13.5)	1.351 (0.812–2.249)	0.247
Re-interventions	76 (27.0)	88 (21.3)	1.363 (0.957–1.939)	0.086	64 (27.8)	49 (21.3)	1.424 (0.929–2.184)	0.105
Recovery								
Hospital stay, days	15 (11–28)	12 (9–18)	—	*<0.001* ^†^	15 (11–29)	12 (9–17)	—	*<0.001* ^†^
ICU stay, days	2 (1–6)	2 (1–4)	—	*<0.001* ^†^	3 (1–8)	2 (1–8)	—	0.068†
Readmission	127 (17.0)	59 (14.3)	1.050 (0.684–1.611)	0.823	35 (15.2)	33 (14.3)	1.071 (0.640–1.794)	0.793
Pathology								
R0	271 (96.1)	396 (95.9)	1.058 (0.488–2.293)	0.887	220 (95.7)	218 (94.8)	1.211 (0.513–2.861)	0.663
Lymph nodes	20 (15–25)	14 (10–19)	—	*<0.001* ^†^	20 (16–26)	14 (10–19)	—	*<0.001* ^†^
Positive lymph nodes	0 (0–1)	0 (0–1)	—	0.863†	0 (0–1)	0 (0–1)	—	0.838†

Data are *n* (%), median (IQR) or OR (95% CI). *P*-values < 0.05 are denoted in italic. ASA indicates American Association of Anesthesiologists; CI, confidence interval; ICU, intensive care unit; IQR, interquartile range; OR, odds ratio; THE, transhiatal esophagectomy.

^*^THE used as reference, *P*-value calculated by logistic regression analysis.

^†^Mann–Whitney *U* test.

^‡^Mortality within patients with a severely complicated clinical course.

### Minimally invasive versus open procedures

Out of 749 high-risk TTE patients, 598 underwent a minimally invasive thoracic phase. No significant difference in mortality or morbidity was observed after a minimally invasive compared to open approach (mortality: 5.9 vs. 6.0%, *P* = 0.981). In the high-risk THE group, 167 out of 413 patients underwent a minimally invasive approach. No significant difference in mortality was observed after a minimally invasive compared to open procedure (2.4 vs. 4.1%, *P* = 0.075). However, significantly more overall morbidity (74.5 vs. 54.9%, OR: *P* < 0.001), anastomotic leakage (31.7 vs. 19.9%, *P* = 0.004), pulmonary complications (36.5% vs. 24.8%, *P* = 0.028) and re-interventions (26.9 vs. 17.5%, *P* = 0.026) were observed after minimally invasive THE.

When comparing patients after a minimally invasive TTE (*n* = 308) to open THE (*n* = 235) no significant difference was observed in mortality. Morbidity was significantly higher in the minimally invasive TTE group compared to the open THE group (70.8 vs. 54.9%, *P* < 0.001). Also, patients after a minimally invasive TTE had significantly more pulmonary complications (41.2 vs. 23.4%, *P* < 0.001), more re-interventions (32.1 vs. 17.4%, *P* < 0.001), a longer hospital stay (*P* = 0.004), and a longer ICU stay (*P* = 0.020).

## DISCUSSION

This study shows significantly higher postoperative mortality, (severe) morbidity and failure to rescue rates for high-risk patients, defined as patients with a Charlson Comorbidity Index of 2 or more, compared to low-risk patients after TTE. A similar increase in severe morbidity rates was observed in high-risk patients who underwent THE; however, mortality, failure to rescue rates and general morbidity rates, including pulmonary complications, were not significantly different. Propensity score-matched analysis suggest that, in high-risk patients, TTE is associated with increased postoperative mortality, (severe) morbidity, pulmonary and cardiac complications, increased number of postoperative interventions, and longer times of admission to ICU and overall hospital stay compared to THE. No differences were observed for failure to rescue rates and surgical complications such as anastomotic leak or surgical-site abscesses. Due to the nature of the two surgical approaches, TTE provided a more thorough oncological resection with a higher lymph node yield, however there was no difference in the median number of positive lymph nodes or R0 resection rate. The subgroup analysis, excluding intrathoracic reconstructions, reveals that in high-risk patients following a McKeown esophagectomy a significantly higher mortality, overall morbidity and pulmonary complication rate is reported compared to THE patients.

The optimal surgical management of esophageal cancer has been a topic of discussion for decades.^30^^,^^31^ Early studies reported oncological benefits of a more extensive transthoracic resection and lymphadenectomy, however these studies were initiated in the monomodal treatment era.^2^^,^^4–6^^,^^32^ A recent population based study confirmed a higher lymph node yield, at the cost of an increased morbidity and short-term mortality in patients following TTE compared to THE.^3^ Although some studies reported similar significantly lower mortality rates^33^ and less postoperative pulmonary complications,^2^^,^^13^ most individual studies did not observe any significant effects in postoperative mortality and morbidity rates after THE compared to TTE.^6^^,^^7^^,^^34–38^ In three meta-analyses, comprising 6 to 48 studies, the effect was pooled across studies, revealing a significantly lower mortality after THE compared to TTE.^31^^,^^39^^,^^40^

As for the impact of preoperative clinical condition, several studies have identified a correlation between negative postoperative outcomes and preoperative factors such as: comorbidities^7^^,^^14^^,^^34^^,^^36^^,^^38^^,^^41^ (hypertension, chronic obstructive pulmonary disease and vascular disease), poor functional status,^7^^,^^34^^,^^36^^,^^38^ high ASA scores^7^^,^^36^^,^^38^ and an older age.^14^^,^^34^^,^^36^ Atkins *et al*.^14^ observed that a Charlson comorbidity index score of three or more was significantly associated to increased mortality rates, comparable to the results of the current study.

The original Charlson comorbidity index was developed in 1987 and comprised 19 categories.^10^ Over the years, the index was repeatedly modified and several coding algorithms were created. Deyo’s algorithm is the most commonly used modification.^21^ This algorithm has been validated in esophageal cancer patients and was therefore selected for current study.^11^ The correct identification patients with poor pre-existing condition by the Charlson comorbidity index was confirmed by the fact that these patients had significantly higher ASA scores. In addition, other tools such as the age-adjusted Charlson comorbidity index, which incorporates age as a correction variable,^42^ or frailty index, which measures physiologic reserve,^43^ could be interesting to elucidate the role of age and frailty in further research.

Within the unmatched and matched cohorts significantly more minimally invasive procedures were performed in the TTE group compared to the THE group. It was decided not to incorporate minimally invasive surgery in the propensity score covariates as the minimally invasive nature of TTE is dissimilar to that of THE. Furthermore, many different types of minimally invasive esophagectomy have been performed during the inclusion period. Although significantly more patients were operated minimally invasive in the TTE group, the expected benefit was not reflected by the results. Therefore, the difference that was found is not likely to be attributed to the difference in minimally invasive approach.^15^ This is solidified by the additional analysis, which revealed no significant difference in mortality after minimally invasive or open surgery. On the other hand, it must be noted that minimally invasive surgery was introduced during the study period with a simultaneous increase in intrathoracic reconstructions. An increased morbidity and mortality related to early learning curves is expected and might have influenced the results after TTE.^44^ This may also explain the higher morbidity rate after minimally invasive THE and acknowledged higher leak rate in the total cohort compared with international benchmarking studies.^45^^,^^46^

Although this current study considers a large sample size with validated data collection of a nationwide population, its anonymized nature led to certain limitations. First, any potential inconsistencies within the anonymized data could not be checked or corrected due to privacy regulations. Second, cause of death was not registered and could not be used in the analysis. Third, information on the decision for type of surgery was not provided, particularly whether the Charlson comorbidity index or other risk assessments tools were used in clinical decision-making. Fourth, propensity score matching cannot adjust for any potential unobserved confounders not provided in the dataset such as: history of smoking or alcohol, localization of lymph node metastasis (abdominal or thoracic) and the center’s level of experience, based on number of annual esophagectomies performed. Lastly, the major limitation of the study is the lack of oncological survival data. Therefore, potential long-term oncological benefit of a transthoracic approach could not be assessed. Future data are needed to put the short-term benefits of less postoperative morbidity in perspective of the long-term oncological outcomes.

The findings of this nationwide study show that patients designated as high-risk, assessed by an easy-to-use validated comorbidity index, have a higher short-term complication profile compared to patients with a low comorbidity index. Results of this propensity score matched analysis indicate that, in high-risk patients, the transhiatal approach is associated with lower postoperative morbidity and mortality compared to a transthoracic approach. In daily practice, surgeons can use these insights when the oncological benefits of a transthoracic approach have to be weighed against the better short-term results of the transhiatal approach in high-risk patients.

## ABBREVIATIONS

DUCA, Dutch Upper GI Cancer Audit; TTE, transthoracic esophagectomy; THE, transhiatal esophagectomy

## Financial support

None.

## Supplementary Material

Supplemental_table_1_doac028
