# Supplementary material for: Short-term outcome for high-risk patients after esophagectomy
Source: Dis Esophagus. 2022 Jun 21;36(1):doac028. doi: 10.1093/dote/doac028 (PMC9817823; doi:10.1093/dote/doac028)
Supplement: Supplemental_table_1_doac028 [file supplemental_table_1_doac028.pdf]

Characteristics	Unmatched cohort high-risk n=695			Matched cohort high-risk n=460		
	McKeown n=282	THE n=413	SMD	McKeown n=230	THE N=230	SMD
Age, years	68 (63-72)	70 (64-75)	0.25	69 (64-72)	68 (62-74)	0.01
BMI, kg/m^2	25.9 (23.5-28.7)	26.1 (23.5-29.2)	0.10	26.2 (23.7-28.7)	26.1 (23.2-29.0)	0.01
Gender						
Male	216 (76.6)	327 (79.2)	0.06	178 (77.4)	179 (77.8)	0.01
CCI score						
2	178 (63.1)	251 (60.8)	0.05	143 (62.2)	143 (62.2)	0.00
3	76 (27.0)	98 (23.7)	0.07	61 (26.5)	59 (25.7)	0.02
≥4	28 (9.9)	64 (15.5)	0.17	26 (11.3)	28 (12.2)	0.03
ASA-classification						
I-II	186 (66.0)	240 (58.1)	0.16	146 (63.5)	152 (66.1)	0.05
III-IV	96 (34.0)	173 (41.9)	0.16	84 (36.5)	78 (33.9)	0.05
Tumor histology						
AC	203 (72.0)	350 (84.7)	0.31	182 (79.1)	184 (80.0)	0.02
SCC	64 (22.7)	54 (13.1)	0.25	41 (17.8)	38 (16.5)	0.03
Other	15 (5.3)	9 (2.2)	0.16	7 (3.0)	8 (3.5)	0.03
Tumor location						
Distal*	241 (85.5)	267 (64.6)	-	192 (83.5)	158 (68.7)	-
GEJ*	41 (14.5)	146 (35.4)	-	38 (16.5)	72 (31.3)	-
From the incisors, cm	34 (31-36)	36 (34-38)	0.59	35 (32-37)	35 (33-37)	0.09
cT-stage						
cT0-1	20 (7.1)	23 (5.6)	0.06	13 (5.7)	12 (5.2)	0.02
cT2	46 (16.3)	92 (22.3)	0.15	42 (18.3)	48 (20.9)	0.06
cT3-4	205 (72.7)	277 (67.1)	0.12	164 (71.3)	163 (70.9)	0.01
cTx	11 (3.9)	21 (5.1)	0.06	11 (4.8)	7 (3.0)	0.09
cN-stage						
cN0	99 (35.1)	185 (44.8)	0.20	89 (38.7)	89 (38.7)	0.00
cN1	112 (39.7)	149 (36.1)	0.07	87 (37.8)	84 (36.5)	0.03
cN2-3	55 (19.5)	60 (14.5)	0.13	41 (17.8)	45 (19.6)	0.05
cNx	16 (5.7)	19 (4.6)	0.07	13 (5.7)	12 (5.2)	0.02
Neoadjuvant therapy						
None	30 (10.6)	69 (16.7)	0.18	26 (11.3)	28 (12.2)	0.03
Chemotherapy	15 (5.3)	33 (8.0)	0.10	12 (5.2)	17 (7.4)	0.08
CRT or radiotherapy	237 (84.0)	311 (75.3)	0.22	192 (83.5)	185 (80.4)	0.08
Previous abdominal or thoracic surgery						
Yes	113 (39.9)	170 (41.2)	0.02	137 (59.6)	135 (58.7)	0.02
Approach*						
Open	54 (19.1)	246 (59.6)	-	40 (17.4)	138 (60.0)	-
MI abdomen	10 (3.5)	167 (40.4)	-	9 (3.9)	92 (40.0)	-
MI thorax	4 (1.4)	-	-	3 (1.3)	-	-
MIE	214 (75.9)	-	-	178 (77.4)	-	-
Anastomosis*						
Cervical	282 (100)	413 (100)	-	230 (100)	230 (100)	-

Supplemental table 1. Baseline characteristics of high-risk after McKeown and THE before and after propensity score matching. Data are n (%) or median (IQR).

AC indicates adenocarcinoma; ASA, American Association of Anesthesiologists; BMI, body mass index; CCI, Charlson Comorbidity Index; CRT, chemoradiotherapy; GEJ, gastroesophageal junction; IQR, interquartile range; MI, minimally invasive; MIE, minimally invasive esophagectomy; SMD, standardized mean difference; SCC, squamous cell carcinoma; THE, transhiatal esophagectomy.

*Variables were not used in propensity score matching
